# Attitudes of sperm, egg and embryo donors and recipients towards genetic information and screening of donors

**DOI:** 10.1186/s12978-018-0468-9

**Published:** 2018-02-09

**Authors:** David J. Amor, Annabelle Kerr, Nandini Somanathan, Alison McEwen, Marianne Tome, Jan Hodgson, Sharon Lewis

**Affiliations:** 1 Murdoch Children’s Research Institute, Royal Children’s Hospital, Parkville, Australia; 2 Department of Paediatrics, The University of Melbourne, Parkville, Australia; 3 Melbourne IVF, East Melbourne, Australia; 4 Graduate School of Health, University of Technology, Sydney, Australia

**Keywords:** Donor sperm, Donor conception, Assisted reproduction, Genetic screening

## Abstract

**Background:**

Gamete and embryo donors undergo genetic screening procedures in order to maximise the health of donor-conceived offspring. In the era of genomic medicine, expanded genetic screening may be offered to donors for the purpose of avoiding transmission of harmful genetic mutations. The objective of this study was to explore the attitudes of donors and recipients toward the expanded genetic screening of donors.

**Methods:**

Qualitative interview study with thematic analysis, undertaken in a tertiary fertility centre. Semi-structured in-depth qualitative interviews were conducted with eleven recipients and nine donors from three different cohorts (sperm, egg and embryo donors/recipients).

**Results:**

Donors and recipients acknowledged the importance of genetic information and were comfortable with the existing level of genetic screening of donors. Recipients recognised some potential benefits of expanded genetic screening of donors; however both recipients and donors were apprehensive about extended genomic technologies, with concerns about how this information would be used and the ethics of genetic selectivity.

**Conclusion:**

Participants in donor programs support some level of genetic screening of donors, but are wary of expanding genetic screening beyond current levels.

## Plain English summary

A relatively small proportion of pregnancies are achieved with the assistance of sperm donors, egg donors or embryo donors. In most assisted reproductive clinics, donors or sperm, eggs or embryos undergo some genetic screening procedures in order to maximise the health of donor-conceived offspring. Recent advances in genetic testing technologies mean that it is now possible to perform more extensive genetic screening of donors than previously was possible.

In this study we conducted in depth interviews with sperm, egg and embryo donors, and with recipients of donor sperm, eggs or embryos, to explore their attitudes towards the collection and use of genetic information in the donor process, and towards the possibility of conducting more extensive genetic screening of donors. Donors and recipients all acknowledged the importance of genetic information and were comfortable with the existing level of genetic screening of donors. Recipients recognised some potential benefits of performing more extensive genetic screening of donors; however both recipients and donors were apprehensive about extended genomic technologies, with concerns about how this information would be used and the ethics of genetic selectivity. The study concludes that participants in donor programs support some level of genetic screening of donors, but are wary of expanding genetic screening beyond current levels.

## Background

Individuals who donate gametes (eggs or sperm) and couples who donate embryos undergo genetic screening procedures that are designed to maximise the health and welfare of donor conceived children [1]. Practices vary between clinics, but typically include two main components: medical and family history of the donor and genetic screening tests. The medical and family history of the donor is designed to exclude the presence of major mendelian disorders, chromosome rearrangements and multifactorial disorders that have a significant genetic component [2, 3]. Genetic tests undertaken in donors may include karyotyping and genetic screening for the carrier status of specific conditions such as cystic fibrosis, spinal muscular atrophy, haemoglobinopathies, Tay-Sachs disease and Fragile X syndrome [2, 4, 5].

Despite implementation of these practices, it is inevitable that serious inherited conditions will, on occasion, occur in donor-conceived children or in individuals who have been donors in the past. Reporting of such instances has prompted calls for more extensive genetic screening of donors [6–8], and advances in genetic testing technology have now provided the opportunity for more expanded genetic screening of gamete and embryo donors [9–11]. To date, most attention has been directed towards expanded carrier screening for autosomal recessive disorders [2, 12, 13], although new genetic testing technologies could potentially be used to screen for undiagnosed autosomal dominant disorders and even for susceptibility to some multifactorial diseases [14].

There is little information about the attitudes of gamete and embryo donors and recipients towards donor genetic screening. A recent on-line survey of women who had used donor sperm found support for the implementation of more comprehensive genetic screening of donors [15]; however there are also concerns amongst health professionals about the effectiveness of expanded genetic screening protocols and the need to treat donors as ‘interested stakeholders, not merely as providers of genetic material’ [2]. In this research we aimed to explore the experiences and attitudes of gamete and embryo donors and recipients towards current donor genetic screening practices and towards potential future expanded donor genetic screening.

## Methods

### Study setting - Melbourne IVF donor program

This research was conducted in the setting of the donor program of Melbourne IVF, a large IVF provider based in Victoria, Australia. In Victoria, the donation of reproductive tissues must be altruistic, and it is illegal for donors to profit from their donation. In addition, a donor-conceived person is entitled to access identifying information about their donor. The Melbourne IVF donor program provides a service whereby people can donate, or be recipients of, gametes (eggs or sperm) and embryos. Donated embryos are from couples and individuals who have undergone IVF treatment and have completed their families. Donor program criteria stipulate that at the time of donation, sperm donors must be aged between 25 and 46 years and egg donors must be aged between 25 and 40 years. Embryos can be donated if the egg donor was aged less than 42 years at the time of embryo creation. All donors must be Australian citizens. Recipients of donor sperm must be aged less than 46 years and recipients of donor eggs or embryos must be aged less than 51 years.

At Melbourne IVF, donors complete a Genetic Health Questionnaire that collects information about personal and family history of conditions with a suspected genetic contribution. In addition to the genetic health questionnaire, donors undergo genetic screening to identify genetic abnormalities that could be transmitted to children that are conceived with their gametes or embryos. Screening comprises a standard karyotype (looking for structural rearrangements) and testing for three common single gene disorders: thalassemia, spinal muscular atrophy and cystic fibrosis. Female donors are also tested for Fragile X syndrome.

### Participants

Two groups of participants were invited to participate in this study.

*Recipients* comprised recipients of gametes (sperm or eggs) and recipients of donor embryos who:

- had used received IVF treatment at Melbourne IVF using donor gametes or embryos between 2012 and 2014
- were not currently pregnant or undergoing IVF treatment
- had not used a genetically related person as a donor
- could speak English.

Demographic data about recipients, including whether or not a child had been born as a result of the donation, were not collected in order to preserve confidentiality.

*Donors* comprised donors of gametes (sperm or eggs) and embryo donors who:

- had donated between 2012 and 2014
- had not donated to a genetic relative
- could speak English.

Demographic data about donors, including whether or not a child had been born as a result of their donation, were not collected in order to preserve confidentiality.

Eligible individuals were contacted by phone and asked if they would like to receive information about the study; an invitation pack was sent to those who indicated interest. If no response was received from participants after two weeks, a reminder was sent. Once a consent form was received by the study team, the participants were contacted to arrange a convenient time to participate in the study. The most recently seen patients were contacted first and recruitment continued until thematic saturation had been reached.

### Methodology

A qualitative methodological approach was employed in this research in order to enable exploration of the experiences of gamete donors and recipients in relation to genetic screening.

### Interviews

A semi structured interview schedule was developed to address the specific research aims for each group (Table 1). It included an exploration of the background to being a donor or recipient, experiences of the health questionnaires, perceptions of important information transfer and participant attitudes to genetic information and genetic testing. Open ended questions were used to allow participants to take the discussion in any direction while maintaining focus of the topic under investigation. Interviews were conducted either face to face or by phone during a six month period in 2015. Interviews of donors were conducted by author NS and interviews of recipients were conducted by author AK. All interviews were digitally recorded.

**Table 1:** Interview Schedule used for donors and recipients

	Areas to address	
Domain	Donors	Recipients
Introduction	− Why the participant became a gamete or embryo donor	− Why the participant(s) required a gamete or embryo donor
Expectations about information	− Expectations of donor program − What information donors thought they would provide to potential recipients	− Expectations of recipient program − What information the recipients thought would be provided about potential donors
Experience of program regarding genetic information (obtained from the donor Genetic Health Questionnaire)	− Thoughts after seeing questionnaires − Unexpected information required − Donors experience with the questionnaires − How the donors thought the information would be used by recipients	− Thoughts after seeing completed questionnaires − Unexpected information given − More/less information than previously thought − How recipients used the donor information
Importance of genetic information	− What information donors thought would be most important to recipients − Medical − Genetic − Personal attributes	− What information was the most important to recipients and why − Medical − Genetic − Personal attributes − Did this remain consistent throughout the recipient process
Attitudes towards genetic information	− Thoughts on need for genetic information about donor − Options of further genetic screening − Personal and family implications	− Thoughts on need for genetic information about potential donors − Options for further genetic screening
Any other information required	− Any other information donors feel could have been provided to recipients	− Any other information recipients feel could have been provided about potential donors

### Analysis

Digital recordings were transcribed verbatim using ExpressScribe Software (NCH Software, Inc., Greenwood Village CO, USA). Transcripts were de-identified and pseudonyms assigned. Transcripts were imported into NVivo Software (QSR International PTY Ltd., Melbourne, Australia) and analysed using thematic analysis. This involved a rigorous process of coding to identify differences and similarities in order to develop themes from within data [16]. A constant comparative approach was used - coding began immediately and continued throughout recruitment so that themes identified early in the process could be further explored with participants in later interviews [17]. The initial stages of coding were performed by NS and AK. The coding and categorizing of data were confirmed through co-coding by SL and AM.

### Ethics committee approval

This study was approved by the Human Research Ethics Committee of Melbourne IVF, Victoria, Australia, reference number 39/15-MIVF.

## Results

### Response

#### Recipients

Thirty gamete/embryo recipients were identified from the MIVF database. Twenty-five gamete/embryo recipients were contacted by phone and 23 agreed to be sent an information pack. Nine recipients returned the consent forms and were interviewed, giving a participation rate of 39%. The recipient participants included three sperm recipients, three egg recipients and three embryo recipients. Two recipients were interviewed with their partner, resulting in a total of 11 participants.

#### Donors

Thirty gamete/embryo donors were identified from the Melbourne IVF database. All were contacted and agreed to be sent an information pack. Eleven donors returned consent forms and nine were available to be interviewed, giving a participant rate of 30%. The donor participants comprised three sperm donors, three egg donors and three (female) embryo donors. Participant demographics are shown in Table 2.

**Table 2:** Demographics of participants

Participants (pseudonyms)	Donation	Relationship status
*Recipients*		
Cathy	Sperm	Different sex
Leonie and Cassie	Sperm	Same sex
Katrina	Sperm	Different sex
Anne	Egg	Different sex
Charlotte	Egg	Different sex
Lucy	Egg	Different sex
Jane	Embryo	Single
Kate	Embryo	Different sex
Dianna and Luke	Embryo	Different sex
*Donors*		
Anthony	Sperm	
Ethan	Sperm	
Euan	Sperm	
Paige	Egg	
Charlotte	Egg	
Becky	Egg	
Paula	Embryo	
Naomi	Embryo	
Lorna	Embryo	

#### Recipient themes

**Fig. 1:**
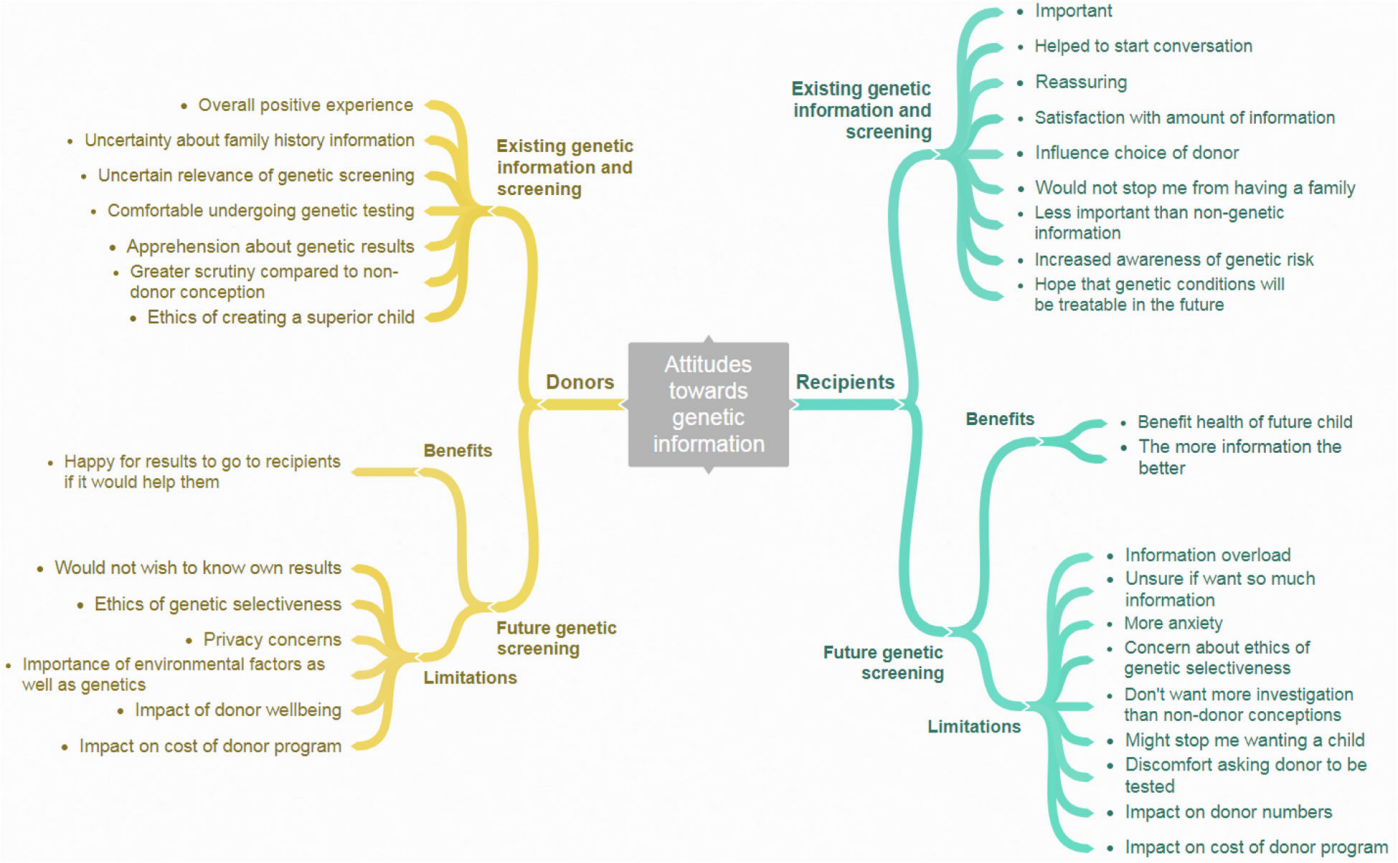
Attitudes of donors and recipients towards genetic information. Themes were identified from participant interviews

Recipient themes are summarised in Fig. 1 and fall into two main categories.

A) *Existing genetic information and screening*

Recipients felt that donor genetic information was important and helpful information to receive during the donor program.

*“Oh yeah, definitely, if you didn’t know that then it is this world wide of unknown of what might happen in your child’s future it’s definitely something important that you need to know that” -*Cathy, sperm recipient.

An egg recipient explained that the genetic information normalised the need to discuss the information with their known egg donor.

*“The donor program is wonderful because it normalises that you need to look for that so it could feel quite intrusive like you are willing to give me a gift and now I’m going to give you the third degree and what’s your health like”-*Anne, egg recipient.

Sperm recipients explained that it helped in the selection of their donor.

*“Yeah, it definitely impacted I think from what we saw on the form, it sort of helped us narrow it down to another couple of donors”*-Leonie, sperm recipient.

While genetic information was important, for many recipients it did not play as significant a role as non-genetic information when selecting a donor.

*“I guess whilst all the genetic information was kind of necessary the other side [non genetic information] was far more important”*-Cassie, sperm recipient.

Recipients mentioned their hope that medical advances would mean that in the future many genetic conditions would be treatable.

*“I was reasonably comfortable to take that risk and I think also 20 or 30 years down the track hopefully something would have come up”*-Jane, embryo recipient.

Recipients explained that if they or their partner had a family history of a genetic condition, it wouldn’t stop them from having a family.

*“When you meet someone …you don’t sort of ask all the genetic information”*-Leonie, sperm recipient.

Four recipients used donors who had a significant genetic or family history, and felt the information was helpful but did not influence their choice of donor.

*“There was a letter saying that there is an increased chance that she could develop hearing loss, but we went with it because we thought well you know these days they do a lot with hearing”*-Cathy, sperm recipient.

Recipients acknowledged that no donor was likely to be free of any genetic risk, and were satisfied with the amount of donor genetic information they received.

*“The chances of getting someone with a perfectly clean medical history is just negligible we are all going to die from something whether it be a stroke, cancer, heart disease, so let’s face it, when you put it on paper and it looks scary but that is the reality for all of us”*-Jane, embryo recipient.

*“For me I was happy with the knowledge that I had”*-Anne, egg recipient.

The feeling of being reassured by the genetic screening process was a common theme among recipients.

*“I actually found that to be very, very, very reassuring”*-Cassie, sperm recipient.

Recipients mentioned that the genetic information provided an awareness of the risks for their future children.

*“It reassured me because it meant that the medical team had looked at it and reviewed it and had given me their opinions and percentages so I could make an informed decision”*-Charlotte, egg recipient.

Embryo recipients expressed how they had not previously thought about genetic information but they were glad they had received it.

*“You know what was really good? Even though I hadn't thought of those things, I’m glad the questionnaire was there”*-Lucy, egg recipient.

B) *Future genetic screening*

Recipients discussed future genetic testing technologies that might be used in the donor program and expressed that more information would be better in their situation.

*“But yeah I certainly think the more information the better”*-Jane, embryo recipient.

However the risk for too much information was also highlighted.

*“You don’t want to have information overload I think”*-Lucy, egg recipient.

Recipients from all cohorts expressed their interest in having the option of more genetic information and found it difficult to determine if it would be wanted or not.

*“It is a really hard one to know whether we would really have wanted more information”*-Kate, embryo recipient.

*“So it’s not as important to me, but I think the option should be there”*-Charlotte, egg recipient.

Recipients stated that more genetic information could benefit many aspects of the donor program. One was the wellbeing of the donor-conceived child.

*“If you had more information it would be positive not only from the selection point of view but from a management point of view in the future”*-Jane, embryo recipient.

Recipients went on to discuss potential adverse consequences from the introduction of new genetic technologies into the donor program. Ethical concerns were raised about choosing donors based on their genetic information, which they interpreted as a form genetic selectivity, or the creation of “designer” babies.

*“It would have given me pause for thought just because it kind of…it sounds very much like it’s starting to create designer babies”*-Charlotte, egg recipient.

Recipients also felt that natural conceptions did not involve this amount of genetic investigation so questioned why donor conceived pregnancies should.

*“You know it is what it is, you know, so it is the risk you take even if you are having your own children you don’t know what is going to come”*-Kate, embryo recipient.

Recipients also viewed extra donor genetic investigation as potentially unhelpful.

*“I think that if we looked into it too much it might stop me from wanting a child”*-Katrina, sperm recipient.

The potential of expanded genetic screening to cause anxiety was recognised by recipients, particularly when discussing the possibilities of knowing about uncertain genetic risks.

*“For me if it was uncertain I would rather not know… and then it’s not in the back of your head and you don’t worry about it”*-Cathy, sperm recipient.

Egg and embryo recipients were concerned about the impact that expanded genetic screening would have on donor numbers, with the majority claiming that they would much rather have more donors than more information.

*“I don’t think there is anything wrong with the information we have now so if I had the choice of more chance of a donor and less information or more information and less chance of a donor I would probably go with the information that I have now which is the current information and an increased number of donors”*-Lucy, egg recipient.

Egg recipients were particularly concerned about the donors’ wellbeing and thoughts on further screening.

*“And then making a donor go through genetic screening because then…say by the way you have made the offer but I want you to go get screened”*-Charlotte, egg recipient.

Egg recipients also felt that further genetic screening would be something they would find difficult to ask the donors to undergo.

*“I’m not genetically screening myself, why would I do that to someone else?”*-Charlotte, egg recipient.

#### Donor themes

Donor themes are summarised in Fig. 1 and fall into two main categories.

A) *Existing genetic information and screening*

The donors understood how important their medical and genetic information was for recipients and found the experience of providing this information to be a positive one. Donors mentioned the difficulty of having to find out their own family history, especially as some of the donors had not told their family members of their choice to donate. There were many factors identified within the genetic screening process and the providing of genetic information and these are outlined below.

Donors felt comfortable answering the medical questions. A few of the donors needed further testing due to a genetic condition being identified in the family history. These donors questioned the relevance of this process, partly because the genetic condition was in a distant family member.

*“I was surprised that I then had to provide a lot more information about that, when…it was in relation to a family member and not in relation to me”* -Euan, sperm donor.

Donors described their difficulty providing family history information. Donors had to ask other family members to help provide genetic histories, and some found the process very interesting, in terms of gathering all the information together.

*“I found it good and I found it hard and sometimes you don’t take notice of what happens in your grandparents or you know what I mean”*-Paige, egg donor.

While all donors had to find out more about their family histories, many struggled to know what to write down on the Genetic Health Questionnaire, and were unsure if certain health conditions identified in their families were genetic or not.

*“And it makes you think about things you do hear, whether they are genetic, and you feel, should I write this down, or shouldn’t I…”*-Naomi, embryo donor.

When asked about the importance of testing, there was a mixed response. Donors did not always see the relevance of genetic screening, but recognised that screening was important, especially if it could prevent the passing on of a serious genetic disease.

*“Personally I don’t see the relevance in it”*-Becky, egg donor.

*“Well, I don’t have an issue with it. It’s safer to know if you have an issue and to not donate, than to accidently pass on a genetic disease”-*Charlotte, egg donor.

Although donors did not consistently recognise the importance of genetic screening tests, all were comfortable undergoing the tests. Donors mentioned their apprehension about finding out the results of their tests, even though they knew of no genetic condition in the family. They felt this was a normal anxious response to having a medical test.

*“There is always that thing of ah discovering stuff you don’t know about…that can be both good and bad at the same time”*-Anthony, sperm donor.

The donors spoke about the level of scrutiny involved in genetic screening, and drew the comparison with natural conception: they noted that a couple conceiving naturally would not need to undergo genetic screening. Donors also expressed opinions about the ethics of genetic screening and whether this type of screening was creating a child that could be superior to a naturally conceived child.

*“I mean I felt like they were trying to create a super human or something... It is a little off putting, that children, that people are selecting their children”-*Euan, sperm donor.

B) *Future genetic screening*

Donors spoke about the future of genetic screening within donor programs. The participants expressed concerns about the ethics of expanded genetic screening in a donor program and whether this might lead to genetic selectivity. One participant compared this type of genetic screening to the movie ‘Gattaca’. Participants raised the concept of the relative roles of genetic and environmental factors in child health, and thought that environmental factors play a greater role than genetics in a child’s development.

*“If it was me and the child was in my house, I would be providing the growing situation and buying the books, so I can influence what that person says more than their sperm donor”*-Ethan, sperm donor.

The level of scrutiny in genetic screening lead many participants to voice their thoughts about the ethics of genetic selectivity and referred to children resulting as “designer babies”. Participants felt that a high level of screening would promote the idea of the perfect child, and felt uncomfortable with the idea of selectivity, which they associated with a higher level of screening.

*“…and the scary thing is then we can choose…choose what sort of babies we are going to have and that’s scary to me”*-Becky, egg donor.

*“Um, it would have given me pause for thought just because it kind of…it sounds very much like it’s starting to create designer babies”*-Charlotte, egg donor.

Most donors, if required to go through this level of screening, would not want to know their results. The participants felt that the knowledge gained from these results would adversely affect their life and personal choices, especially if a detrimental disease was discovered. However, while the participants did not want to know their result, they were happy for the results to be given to recipients if it would help them choose a donor. Participants spoke about specific genetic diseases that have incomplete penetrance. These participants felt that knowing such a result would give them a lifelong fear and would leave them struggling to plan a future. However one participant also worried about the laboratory scientists knowing her information and what that would mean for her privacy.

“*When you find out that your children have predispositions to illnesses and maybe if you don’t know it could be…I don’t know if it’s good to know that much…”*-Naomi, embryo donor.

*“I don’t think I would want those results personally but I don’t mind if other people have those results or that it’s tested for”*-Ethan, sperm donor.

*“But, then the scientists are going to know this information about me”*-Becky, egg donor.

Participants questioned whether society was "crossing a line" by trying to create perfect human beings. Morally they felt this type of screening was wrong and would create inequality, especially for those who were conceived naturally. There was a general feeling that technology has advanced very quickly but the science is still lacking and more thought should be given as to whether these testing procedures could do more harm than good.

*“There would be things that you would need to draw the line somewhere, things that are controllable and things that aren’t controllable, to not create a society which is too bound by perfecting itself”*-Charlotte, egg donor.

Donors were also concerned that an increased level of genetic screening would affect whether individuals would choose to donate. These participants thought they would still donate if expanded screening was introduced; however many of these participants would not want to know their own results. Participants worried about the impact this level of screening would have on their lives and thought that more thought should be given into whether this level of screening is ethical or beneficial.

*“I think less people would [want to donate]. I mean…um…we all have a little something in our closet that we don’t want to share with others”*-Becky, egg donor.

## Discussion

This qualitative study explored attitudes towards the genetic screening of gamete (sperm or egg) and embryo donors, from the dual perspectives of the donors and the recipients, providing valuable information that will assist in the design and implementation of donor programs in the context of the availability of expanded genetic screening. All donors and recipients had personal experience of the donor screening process at Melbourne IVF, which comprised a Genetic Health Questionnaire and limited genetic testing.

In relation to the Genetic Health Questionnaire, all donors and recipients understood the importance of the information contained in the questionnaire. However some donors had difficulty filling out the questionnaire because they were uncertain about aspects of their family history, and about whether or not certain medical conditions in their family history were genetic in origin. While the questionnaire is a cost effective way of gathering family history information, our results suggest that the process could lead to misinformation, and a face-to-face interview may be more effective. Recipients appreciated and valued the information provided by donors in the Genetic Health Questionnaire, but also felt that when selecting a donor, this information was less important than non-health related information, such as physical attributes, values and beliefs, and character descriptors. Most recipients did not feel that they would reject a donor based on family medical history information alone. Although previous studies have not addressed attitudes towards genetic information, our results are comparable to a previous Australian study which showed that health information was ranked as the most important type of donor information by sperm and egg recipients and by egg donors; sperm donors ranked health information as the second most important form of donor information, behind donor physical characteristics [18].

Donors and recipients supported the existing level of genetic testing in the donor program, which comprises karyotype and carrier screening for thalassemia, spinal muscular atrophy, cystic fibrosis and Fragile X syndrome. Some donors questioned the relevance of these tests but nonetheless were happy to be tested. However when asked about the opportunity for expanded genetic testing of donors, donors and recipients had reservations and questioned the desirability of expanded genetic screening. A specific concern expressed by donors and recipients alike was that choosing donors based on the results of expanded genetic screening might represent an undesirable move towards genetic selectivity, which they likened to having a "designer baby". In addition, donors and recipients expressed concern that increased genetic screening might deter donors from donating, resulting in a decline in donor numbers.

Donors also expressed concern about the psychological impact of them receiving this additional genetic information. Interestingly, some donors stated that although they would consent to expanded genetic screening being performed in them, they would not wish to be informed of the results. In practice, IVF clinics are unlikely to agree to such a request, as it would place both the clinic and the recipient in the position of holding undisclosed genetic information that might be important for the health of the donor. Recipients were concerned that expanded genetic screening of donors might cause increased anxiety to recipients, as well as increasing the financial cost of accessing the donor program. Although many recipients recognised potential benefits of having more genetic information about donors, such as an opportunity to maximise the health of their donor-conceived child, they were uncertain whether they would actually want this information themselves. In addition, participants highlighted the important contribution of environmental factors to child health, and did not view child health as being determined only by genetics.

No recipients were strongly in favour of receiving extended screening and genetic information about donors. Although attitudes towards extended carrier screening have not been studied in a donor population, reticence about the extended carrier screening has previously been observed amongst health professionals and patient representatives, with particular concerns centring on the ethical and psychological challenges of expanded carrier screening [9, 19, 20].

Genetic information was viewed differently by recipients of donor sperm compared to recipients of donor eggs/embryos. In particular, whilst genetic information was viewed by recipients of donor sperm as a factor in their selecting a sperm donor, recipients of donor eggs or embryos mostly saw genetic information as something to be passed on to their offspring. This was partly because recipients of donor eggs or embryos had less opportunity to choose between alternative donors, and because egg donors were known to their recipients. Egg recipients in particular reported not having considered the genetic history of the donor before they were provided with the genetic health questionnaires.

Overall, this research has highlighted that while donors and recipients are supportive of existing genetic screening of gamete and embryo donors, they have reservations about expanded genetic screening. This was particularly prominent amongst donors, but was also an issue for recipients who were concerned about the impact of expanded genetic screening on the cost and availability of accessing donors. These findings contrast to those of Sawyer et al. [15] who found support amongst recipients for expanded genetic screening of donors and a willingness from recipients to pay extra for this service. Notably, the study by Sawyer et al. was conducted as an on line survey, with 85% of respondents residing in the USA. Differences between the results of the two studies may be due to differences in study methodology, and between donor programs in USA and Australia. In Australia, the perspectives of donors and recipients are likely influenced by the fact that donation of reproductive tissues must be altruistic, and it is illegal for donors to profit from their donation. When interpreting this study, it is also important to note that the number of donors and recipients in each category (egg/sperm/embryo) is small and may not be representative of the donor community as a whole. In addition, the overall participation rate of 34% represents a potential source of bias.

## Conclusion

Participants in our donor program support some level of genetic screening of donors, but have concerns about expanding genetic screening beyond current levels. The implementation of expanded genetic screening in donor programs may do more harm than good.
